# Empowering Thai community pharmacists in combating antimicrobial resistance: Qualitative insight and sentiment analysis

**DOI:** 10.1016/j.rcsop.2024.100535

**Published:** 2024-11-03

**Authors:** Rojjares Netthong, Sisira Donsamak, Dai N. John, Ros Kane, Keivan Armani

**Affiliations:** a Faculty of Pharmaceutical Sciences, Ubon Ratchathani University, Ubon Ratchathani 34190, Thailand; b School of Pharmacy and Pharmaceutical Sciences, Cardiff University, Cardiff, UK; c School of Health and Care Sciences, College of Health and Science, University of Lincoln, Lincoln, UK; d Department of Primary Care and Public Health, School of Public Health, Faculty of Medicine, Imperial College London, UK; e Faculty of Pharmaceutical Sciences, UCSI University, Kuala Lumpur, Malaysia

**Keywords:** Antimicrobial, Antibiotic resistance, Community pharmacy, Pharmacist, Thailand

## Abstract

**Background:**

Antimicrobial resistance (AMR) is increasing globally and poses a significant public health challenge. Community pharmacists, especially in primary care settings, play a pivotal role in mitigating the irrational use of antibiotics, a key driver of AMR.

**Objectives:**

This study aims to explore qualitative insights from community pharmacists regarding antibiotic supply and usage, analyze sentiments related to AMR, and highlight the crucial role of community pharmacists in AMR stewardship at the primary care/community level.

**Methods:**

This study engaged community pharmacists in Thailand through semi-structured interviews to obtain in-depth insights into the antibiotic supply and perceptions of AMR. Additionally, sentiment analysis, which evaluates the emotional tone of the pharmacists' responses, was conducted to enrich the findings.

**Results:**

Interviews with 23 community pharmacists highlighted the practices, challenges, and strategies related to antibiotic supply and use. Key findings include the identification of barriers such as patient demand and lack of awareness about antimicrobial resistance (AMR), alongside strategies for improvement such as public education and professional development. Sentiment analysis reveals a cautiously optimistic perspective toward enhancing rational antibiotic use, underscoring the importance of comprehensive approaches that combine education, ethics, and regulatory measures to address the complexities of antibiotic management at community pharmacies.

**Conclusion:**

This study underscores the necessity of public awareness, pharmacist–patient relationships, and regulatory reforms for the rational use of antibiotics in community pharmacies. These findings emphasize that pharmacist education and adherence to professional ethics are essential for mitigating antimicrobial resistance and promoting rational antibiotic use.

## Highlights

• Key factors influencing antibiotic supply in Thai pharmacies were identified.
• A qualitative and sentimental analysis of the pharmacists' perceptions was conducted.
• Patient demand and commercial pressures challenge rational antibiotic use.
• Gaps in pharmacists' knowledge and regulatory enforcement were highlighted.
• Strategies proposed to enhance antimicrobial stewardship and education.

## 1 Background

Antimicrobial resistance (AMR) represents one of the most pressing public health crises of our time, with its impact felt across the globe.1., 2., 3, 4, 5 The World Health Organization (WHO) is coordinating a campaign to raise awareness of AMR and encourage best practices to avoid further emergence and spread of AMR, as demonstrated by the Global Action Plan on AMR, which was endorsed by the WHO almost a decade ago.^1^^,^^2^^,^^6^ South East Asia, particularly, stands out as a region severely affected by this burgeoning threat, a situation compounded by its unique healthcare challenges and rapid economic development.^7^ These factors contribute significantly to the AMR burden, creating a complex health landscape where traditional approaches to managing infections are increasingly ineffective. In Thailand, the intersection of healthcare system peculiarities and economic factors, such as increased purchasing power and the absence of rigorous antibiotic regulation, has facilitated easy access to antibiotics, further exacerbating the problem of AMR.8., 9, 10, 11 Although AMR is a natural process that occurs when microorganisms adapt and grow in the presence of antimicrobial agents, increasing the utilization and irrational use of antimicrobial agents accelerates the emergence of resistance.^6^^,^12., 13., 14 In addition, the lack of development of new antimicrobial agents may also exacerbate AMR.^12^ There is a significant association between antimicrobial resistance and inappropriate antibiotic use at both the individual and population levels.^1^^,^^15^^,^^16^ The inappropriate use (including overuse or misuse) of antibiotics results not only in an increase in resistant bacteria but also in ineffective therapy, adverse drug reactions, wasted resources, an increase in the cost of therapy and ultimately a greater economic burden on national and global health systems.^17^^,^^18^ This scenario underscores the need for innovative strategies and localized solutions to mitigate AMR spread effectively.

Community pharmacists are uniquely positioned to address this challenge, as they are easily accessible to the public and play a crucial role in healthcare delivery, which has evolved significantly beyond traditional pharmacy practices.^19^ Positioned as the first point of contact for many patients seeking medical advice and medication, community pharmacists are exceptionally placed to influence antibiotic use and stewardship as the vanguard of the battle against AMR.^20^ Their role now encompasses a broad spectrum of activities, including public education, adherence to regulatory guidelines, and active participation in AMR stewardship initiatives. ^20^ Leading pharmacy organizations, including the International Pharmaceutical Federation (FIP), the General Pharmaceutical Council (GPhC), the American College of Clinical Pharmacy (ACCP), and various primary care networks, have highlighted the invaluable contributions of community pharmacists, which include educating the public on appropriate antibiotic use and implementing comprehensive AMR stewardship programs.^21^

In Thailand, community pharmacists are legally able to supply most medicines without prescriptions, including most antibiotics. However, the supply of antibiotics without a prescription is often illegal in many countries. Nevertheless, obtaining antibiotics without a prescription from community pharmacies has been reported. ^21^ This might be because of the ease of access, availability of medicines, shorter waiting times, and longer working hours.^22^ Furthermore, many patients are unable to afford the fees to consult a physician for their medical needs.^23^, ^24^, ^25^ Community pharmacists in Thailand typically triage and supply medicines (without a prescription) to treat minor illnesses and provide necessary advice. Dispensing services for prescription medicines constitute a very small fraction of the services provided by community pharmacies because drug prescribing and dispensing services are not formally separated in Thailand.^26^, ^27^, ^28^ Consequently, an inappropriate supply of antibiotics for common, self-limiting diseases such as upper respiratory tract infection (URTI), acute diarrhea and simple wounds is common. 29, 30, 31 Reports from national antimicrobial surveillance surveys as well as from a previous study revealed that antimicrobial resistance in Thailand has been increasing over the past decade.^26^^,^^27^^,^^32^

Despite the recognized significance of community pharmacists in the AMR mitigation framework, a notable research gap persists, particularly concerning the qualitative insights and sentiments of these healthcare professionals within the Thai context.^11^^,^^18^^,^^29^^,^^30^^,^^33^, ^34^, ^35^ There have not been any published studies exploring why community pharmacists in Thailand supply antibiotics to their clients, particularly those that focus on the practice of supplying antibiotics without a legitimate prescription by a registered physician. This lack of research underscores the need to examine the legal provisions governing the supply of antibiotics in the country, including existing national drug laws and the enforcement challenges that may facilitate inappropriate antibiotic dispensing. This study seeks to bridge this gap through a holistic approach, not only accentuating the pivotal role of community pharmacists in combating AMR but also exploring their experiences, challenges, and perspectives via qualitative insights and sentiment analysis. By incorporating viewpoints from both international and local pharmacy organizations, this study seeks to explore the multifaceted contributions of community pharmacists to AMR mitigation, setting the stage for more effective strategies and policies tailored to the unique healthcare landscape of Southeast Asia and beyond. Understanding why and how Thai community pharmacists supply antibiotics is important for planning strategies to improve the rational use of antibiotics in communities in Thailand. Therefore, this study aimed to explore the factors and barriers that influence the supply of antibiotics from community pharmacies and to identify ways to increase the rational supply of antibiotics in Thailand.

## 2 Methods

### 2.1 Study design

This study utilized a qualitative approach to investigate community pharmacists' practices and perspectives regarding the supply of antibiotics in Thailand. The research utilized face-to-face semi-structured interviews with community pharmacists in Thailand to understand the ‘how’ and ‘why’ behind the antibiotic supply, as well as to learn about their experiences and views regarding antibiotic use. Ethical approval was obtained from the Cardiff University School of Pharmacy and Pharmaceutical Sciences Ethics Committee (project number 512533) and the Research Ethics Committee of Ubon Ratchathani University, Thailand (project number UBU-REC-27/2560).

### 2.2 Interview topic guide development

The interview topic guide was developed by S.D. on the basis of insights from the literature on the supply of antibiotics from community pharmacies.^26^^,^^27^^,^^36^ This process was guided by a framework that included current prevalent practices of community pharmacies related to antibiotic supply, ensuring that the guide was contextually relevant. The guide, which was refined collaboratively by the research team, aimed to explore pharmacists' experiences with antibiotic supply, their views on antibiotics, the AMR challenge, and strategies to mitigate this issue. A pilot test with a small group of community pharmacists was conducted to validate the interview guide and refine the questions on the basis of their feedback. The interview guide was developed in English and then translated into Thai via a meaning-based approach. A Thai–English bilingual translator facilitated this translation, ensuring that cultural nuances were preserved. The Thai version of the guide was then back-translated into English to verify the accuracy of the translation.

### 2.3 Study sample

Using purposive and convenience sampling methods via email and phone invitations, community pharmacists were selected to ensure diverse samples in terms of pharmacy type (independent and chain, rural and urban) and participant demographics (age, gender, education level, and experience). The participants were told that the study would examine and discuss their antibiotic use, experience and passion for antibiotic stewardship to reduce antimicrobial resistance. The study included three provinces in northeast Thailand (Nakhon Phanom, Ubon Ratchathani, and Sisaket) and three in central Thailand (Bangkok, Nonthaburi, Pathum Thani).

### 2.4 Data collection

The interviews were conducted face-to-face by S.D., who has experience and training in qualitative research, prior to capturing firsthand insights into the pharmacists' experiences and viewpoints regarding antibiotic use and supply in community pharmacies at their workplaces and online video conferences in private settings. The participants provided written consent and were assured that they could withdraw at any time without consequences. Field notes were taken during and after the interviews to preserve nonverbal clues and context. The interviews lasted 45–60 min. The interviews, which were conducted in Thai, were audio-recorded, transcribed verbatim, translated into English by a Thai researcher via a meaning-based approach, and anonymized before analysis. A Thai–English bilingual translator verified the translation accuracy.

### 2.5 Data analysis

The data were analyzed through thematic analysis aided by NVivo qualitative data analysis software. The quotations were displayed and categorized by participant number. The analysis employed both deductive and inductive approaches: the former was guided by the study's aims and the interview guide, with a focus on the factors influencing antibiotic use and supply; the latter identified additional themes emerging from the data.

### 2.6 Sentiment analysis integration

Following thematic analysis, sentiment analysis was carried out to assess the emotional tone behind the pharmacists' responses. TextBlob in Python provides straightforward methods to compute these scores.^36^ Sentiment analysis refers to the process of computing the sentiment concerning the text. The sentiment describes the polarity and subjectivity of the text. Polarity measures the sentiment of the whole text. It could vary from −1, which would be perfectly negative/unfavorable, to +1, which would be perfectly positive or favorable. Subjectivity measures how much of a personal opinion, emotion, or belief is expressed in the text containing it. It ranges from 0 (completely objective) to 1 (completely subjective). Neutral sentiment refers to a sentiment that is in the middle of the two poles and is definitely neither positive nor negative. Instead, the text that is neutral has no strong emotions or opinions and represents the information or description of the situation in an objective way.^37^^,^^38^

This analysis aimed to evaluate the expressions of the participants regarding their practices, perceptions of AMR, and effectiveness of current interventions. Sentiment analysis provided an additional layer of insight, complementing the thematic findings by highlighting the underlying emotions that pharmacists associated with AMR challenges and the supply of antibiotics. This combined approach enriches the understanding of pharmacists' perspectives, offering a more refined view of their motivations, concerns, the degree to which their views are favorable, or otherwise, and the potential impact of their practices on AMR.

## 3 Results

Twenty-three community pharmacists were interviewed for a mean duration of thirty minutes so that participants had sufficient time to share their experiences and insights. The majority of the participants were female (18), had a bachelor's degree in pharmacy (21) and were pharmacy owners (18). Table 1 shows the community pharmacists' characteristics. An approximately equal number of rural (10) and urban (13) pharmacists were interviewed and for clarity, quotations from pharmacists were identified as being from either urban or rural settings.

**Table 1:** Characteristics of the interviewed community pharmacists.

Characteristics	N
**Age**	
30 years and younger	5
31–40 years	10
41–50 years	4
51 years and older	4

**Gender**	
Male	5
Female	18

**Highest education**	
BPharm	22
Pharm D	1

**Years of experience**	
1–5 years	9
6–10 years	9
11 years and over	5

**Ownership**	
Nonowner	5
Owner	18

**Type of pharmacy**	
Independent pharmacy	20
Chain pharmacy	3

**Pharmacy location**	
Urban	13
Rural	10

With respect to the supply of antibiotics, pharmacists reported commonly supplying antibiotics for URTIs, acute diarrhea, simple wounds or urinary tract infections. From the thematic analysis, four themes regarding antibiotic supply were identified, namely, (1) the practice of pharmacists regarding antibiotic supply, (2) barriers to comply with the rational use of antibiotics, (3) limitations of the current program to promote rational use of antibiotics to community pharmacists, and (4) suggestions to improve rational use of antibiotics.

### 3.1 Theme 1: the practice of pharmacists regarding antibiotic supply

Community pharmacists outlined where they would supply antibiotics, whether in response to patients presenting at the pharmacy via history taking and/or by patients describing their symptoms, including direct requests by patients. Patients' symptoms and medical history were used to make clinical decisions. In both urban and rural settings, pharmacists highlighted how they assess patients presenting at the pharmacy. In urban areas, where patients often have more access to health information, pharmacists noted a trend of self-diagnosis among patients.

*Usually, when patients come to my pharmacy, I will take their history. Many times, patients came to me and were self-diagnosed. However, after I took their history, it was a different ailment. [Pharmacist 2, male, 49 years old, 15 years*' *experience, urban] Sentiment: Polarity score: 0.1, subjectivity score: 0.6: This text expresses a neutral to slightly favorable sentiment about the pharmacist's thorough approach. This approach is objective in taking patients' histories but also reflects the pharmacist's personal judgment in identifying different ailments.*

In some cases, pharmacists dispensed antibiotics on the basis of what they thought could help the patient. For example, pharmacists contacted antibiotics for patients who had previously been seriously ill, patients who were likely to have serious bacterial infections, or where it was not yet clear whether there was a bacterial infection. Inappropriate criteria, in this case, include indications such as the administration of antibiotics due to a severe sore throat, fever, or badly discolored nasal discharge that do not prove anything to do with treatment. In the following example, the Thai Public Health Ministry and “Antibiotics Smart Use” both stipulate that only strong evidence of bacterial infection is an adequate criterion for administering antibiotics, not the presence of fever or discolored sputum.

*If I am certain that it is not a bacterial infection, I will not supply antibiotics. However, when I am not sure, for example, patients have diarrhea for days and the frequency of diarrhea is still the same, or in cases where patients have diabetes or other conditions, I will supply antibiotics. [Pharmacist 19, female, 30 years old, 6 years*' *experience, urban] Sentiment: Polarity score: 0.05, subjectivity score: 0.7: The text reflects a slightly favorable sentiment toward cautious antibiotic supply, indicating the pharmacist's careful consideration. A high subjectivity score reflects the personal judgment and decision-making process involved.*

Most pharmacists reported supplying antibiotics for 3–5 days. The economic status of patients was the main reason for an incomplete course of antibiotics. A rural pharmacist shared that some patients were receiving incomplete courses of treatment, situation that reflects the practical constraints faced by pharmacists in rural areas, where patient relationships can significantly impact business continuity.

*I couldn*'*t supply [antibiotics] for ten days; five days is OK for me, and a patient accepted that. [Pharmacist 6, female, 27 years old, 3 years*' *experience, rural] Sentiment: Polarity score: 0.1, subjectivity score: 0.65: This text indicates a slightly favorable sentiment toward the compromise reached with the patient, showing practical constraints and acceptance. The subjectivity score is high because of the personal nature of the decision and patient interaction.*

In addition, some interviewees also advised about appropriate treatment as an alternative to antibiotics and/or offered advice on nonpharmacological self-care options.

*If a patient [who requested antibiotics] has a sore throat, I will advise him/her to take traditional medicines,* e.g.*, Kariyat (Andrographis paniculate). [Pharmacist 13, female, 35 years old, 11 years*' *experience, rural] Sentiment: Polarity score: 0.15, subjectivity score: 0.5: The text conveys a slightly favorable sentiment toward recommending traditional medicine as an alternative. The subjectivity score is moderate, reflecting a balance between personal advice and objective recommendations.*

Many participants reported counseling patients about appropriate antibiotic use when patients request inappropriate antibiotics, and pharmacists counsel them. An urban pharmacist noted that pharmacists should provide patients with information to help them make decisions about antibiotic use and its consequences.

*I will explain to them the reason. What are the advantages? What are the disadvantages? This is our [pharmacist] role. If they don*'*t believe us, it*'*s their decision. [Pharmacist 1, female, 42 years old, 8 years*' *experience, urban] Sentiment: Polarity score: 0.2, subjectivity score: 0.6: This text shows favorable sentiment toward patient education and professional responsibility. A high subjectivity score indicates the personal effort and perspective involved in counseling patients.*

The sentiment analysis revealed views that were slightly favorable (polarity of 0.12) and expressive of personal opinions and feelings (subjectivity of 0.61). This finding indicates that the overall sentiment of the text is slightly favorable and moderately subjective. The pharmacists demonstrate a careful and balanced approach to antibiotic supply, with an emphasis on patient education and responsible decision-making, while expressing some personal views as a result of their professional education.

### 3.2 Theme 2: barriers to comply with the rational use of antibiotics

The participants mentioned several barriers that made it difficult for them to comply with the rational use of antibiotics, including patient demand and commercial interest. Additionally, pharmacists' lack of knowledge regarding antibiotic treatments and their understanding of the significance of AMR were also identified.

Patient demand for antibiotics is one of the most important obstacles to compliance with the rational use of antibiotics, as perceived by community pharmacists. Previous successful use of antibiotics, including obtaining antibiotics from physicians, other healthcare providers or pharmacies, is a significant factor for patients seeking antibiotics from a pharmacy. Other sources of information for patients seeking antibiotics were advice from family members and friends, the internet, and illegal direct advertisements from pharmaceutical companies. Illegal direct advertisements from pharmaceutical companies refer to promotional activities that do not comply with regulatory guidelines. An example includes advertisements that directly target consumers with claims about the benefits of antibiotics without proper disclosure of the need for a prescription or potential risks. These practices are not aligned with the legal requirements for pharmaceutical marketing, which aim to ensure that medications are prescribed and dispensed on the basis of clinical needs rather than consumer demand generated by marketing. This pressure was reported more acutely in urban settings, where access to healthcare information is prevalent, resulting in higher expectations for immediate antibiotic supply. In contrast, rural pharmacists may experience patient demand driven by fewer healthcare alternatives, leading to a stronger reliance on pharmacists for medication.

*They had taken these medications [antibiotics], and they were cured, so they thought that if they were sick, they had to take them immediately. [Pharmacist 18, female 34 years old, 1 years*' *experience, urban] Sentiment: Polarity score:* −*0.1, subjectivity score: 0.7: This text reflects an unfavorable sentiment toward patients' misconceptions about antibiotic use. The subjectivity score is high because of the personal opinion and observation of the pharmacist.*

The conflict of interest between the pharmacy profession and the business was a significant barrier to complying with the rational use of antibiotics. A financial profit was a basic expectation of running the business and was acknowledged by most participants, so they sometimes did not comply with the practice guidelines. Pharmacists were keen to keep their regular customers satisfied with their business while working within the law.

*When I ran my pharmacy for the first time, I felt like I wanted to solve the problem [inappropriate use of antibiotics]. However, when I faced real situations of pressure from patients and economics, I had to surrender in some cases. [Pharmacist 4, female, 50 years old, 20 years*' *experience, urban] Sentiment: Polarity score:* −*0.2; subjectivity score: 0.8. This text expresses an unfavorable sentiment due to the pressures faced by pharmacists, leading to an inappropriate antibiotic supply. A high subjectivity score indicates the personal struggle and disappointment of the pharmacist.*

*For those who do not listen to me and insist on having it [antibiotics], I will supply it because if I don*'*t supply them, they will visit another pharmacy. I need to keep them at my pharmacy. [Pharmacist 14, female, 62 years old, 30 years*' *experience, rural] Sentiment: Polarity score:* −*0.3; subjectivity score: 0.9. The sentiment of this text is unfavorable and indicates economic pressure to keep customers even if they misuse antibiotics. A very high subjectivity score suggests highly personal and practical conflict.*

Most participants stated that they did try to advise patients to receive appropriate treatments. However, they also reported that sometimes, they did not have enough time to instruct or educate patients on the appropriate use of antibiotics, especially when there were multiple clients in the pharmacy at once or when the patients were in a hurry. This resulted in pharmacists supplying antibiotics as requested, even sometimes when it was inappropriate.

*It takes time to educate each patient and takes a long time. In the morning, I have multiple clients at once; I don*'*t have time to educate each of them, so I have to supply what they want. If I take longer than five or ten minutes, the patients will rush me. [Pharmacist 13, female, 35 years old, 11 years*' *experience, rural] Sentiment: Polarity score:* −*0.25, subjectivity score: 0.8: This text reflects an unfavorable sentiment due to time constraints and pressure to meet patient demands. The high subjectivity score reflects the personal experience and challenges faced by the pharmacist.*

Many participants stated that they thought that antibiotic resistance in Thailand was a serious problem. However, some of them mentioned that they felt that this problem was distant from community pharmacies. In addition, a few participants stated that they did not need to worry about antibiotic resistance because pharmaceutical companies have been developing new antibiotics to treat those resistant bacteria.

*I think antibiotic resistance is not a big problem. They [other people] think that the inappropriate use of antibiotics from community pharmacies is one of the causes of serious bacterial resistance infections that are found in hospitals. It*'*s totally different. [Pharmacist 23, female, 37 years old, 10 years*' *experience, rural] Sentiment: Polarity score:* −*0.15, subjectivity score: 0.7: This text reflects an unfavorable sentiment toward the perceived disconnect between community pharmacy practices and hospital-based resistance issues. The subjectivity score is high, reflecting the personal opinions and beliefs of the pharmacist.*

*I think it is normal. Thus, antibiotics that have been used for a long period of time will become resistant. It*'*s normal, but they [pharmaceutical companies] have been developing new antibiotics to fight resistant bacteria. [Pharmacist 9, female, 25 years old, 2 years*' *experience, urban] Sentiment: Polarity Score:* −*0.1, Subjectivity Score: 0.6: This text reflects a slightly unfavorable sentiment, accepting resistance as normal but relying on pharmaceutical companies to address it. The subjectivity score is moderate, reflecting a mix of opinions and factual statements.*

A lack of appropriate knowledge regarding antibiotic treatment was identified as a factor for the provision of irrational antibiotics by community pharmacists. Many community pharmacists mentioned inappropriate key criteria to supply antibiotics. Some pharmacists provide antibiotics for sore throat patients with fever and discolored nasal discharge or sputum or those whose symptoms do not improve with other medicines. Some pharmacists provide antibiotics to patients with acute diarrhea and fever. In addition, some pharmacists believe that the use of antibiotics overall reduces the cost and duration of treatment; thus, patients can return to normal life more quickly and are relieved of symptoms more rapidly. For this reason, pharmacists were also driven to supply antibiotics.

*I usually ask patients about the duration of their illness and previous medications. If patients have sore throat, phlegm, or colored discharge, their symptoms last for 4–5 days, they have already taken other medicines, but they do not feel better, and I will supply antibiotics. [Pharmacist 21, female, 38 years old, 5 years*' *experience, rural] Sentiment: Polarity score:* −*0.05, subjectivity score: 0.5: This text reflects a slightly unfavorable sentiment toward the cautious approach in antibiotic supply on the basis of specific symptoms. The subjectivity score is moderate, reflecting a balance between personal judgment and clinical decision-making.*

Many pharmacists also noted that AMR was the responsibility of others, that is, other healthcare providers and patients, not community pharmacists. Most inappropriate antibiotics are prescribed by doctors, especially from private clinics and hospitals. Inappropriate use of antibiotics due to patient noncompliance can also cause AMR.

*Antimicrobial resistance is not related to pharmacies. This is related to patients*' *behavior, which led to the old package of antibiotics and asked for them. AMR is a small problem. Pharmaceutical companies have already been preparing new antibiotics. [Pharmacist 3, male, 53 years old, 18 years*' *experience, urban] Sentiment: Polarity score:* −*0.2, subjectivity score: 0.7: This text reflects an unfavorable sentiment, downplaying the role of pharmacies in AMR and shifting the responsibility to patients and pharmaceutical companies. A high subjectivity score reflects personal beliefs and opinions.*

*I think that supplying antibiotics from pharmacies is most appropriate. We [community pharmacists] supply only basic antibiotics for minor ailments. Inappropriate use of antibiotics, mostly from doctor clinics, is recommended. [Pharmacist 7, male, 58 years old, 7 years*' *experience, urban] Sentiment: Polarity score:* −*0.1, subjectivity score: 0.6: This text reflects a slightly unfavorable sentiment toward the perceived appropriateness of antibiotic supply in pharmacies while attributing inappropriate use to other healthcare settings. The subjectivity score is moderate, reflecting personal opinions.*

In examining the barriers to adhering to the rational use of antibiotics, the sentiment analysis of the provided text yields a subjectivity score of 0.71 and a polarity score of −0.18. These findings suggest that the text maintains a high degree of subjectivity and conveys an overall sentiment that is unfavorable. The pharmacists highlight various pressures and misconceptions that lead to inappropriate antibiotic use, emphasizing the challenges they face in balancing professional responsibilities with practical constraints.

#### 3.2.1 Theme 3: limitations of current campaigns to promote the rational use of antibiotics in community pharmacies

From the viewpoint of community pharmacists, the current campaign [Rational Drug Use campaign] was not publicized appropriately, so there was a lack of awareness. They also indicated that there were no incentives to participate, nor were there any penalties for not participating in the campaign.

*I never heard about the current campaign. I heard about the promotion of using antibiotics appropriately only when I was studying [at the university]. [Pharmacist 4, female, 50 years old, 20 years*' *experience, urban] Sentiment: Polarity score:* −*0.15; subjectivity score: 0.6: This text reflects an unfavorable sentiment toward the awareness and dissemination of current campaigns, highlighting a gap in communication. The subjectivity score is moderately high, reflecting the personal experience and perspective of the pharmacist.*

*There is no direct impact on the pharmacies [toward the appropriate or inappropriate supply of antibiotics]. For public health facilities, there are key performance indicators (KPIs), which are related to funding from the government. [Pharmacist 21, female, 38 years old, 5 years*' *experience, rural] Sentiment: Polarity score:* −*0.1, subjectivity score: 0.5: This text reflects an unfavorable sentiment regarding the lack of campaigns*' *direct impact on pharmacies. A contrasting point is made for public health facilities that have KPIs related to funding from the government. The subjectivity score is moderate, as the text reflects both personal observations and factual information.*

The polarity and subjectivity scores, derived from sentiment analysis of the text, are −0.13 and 0.55, respectively. These results suggest that the text possesses a moderate level of subjectivity and conveys an overall sentiment that is unfavorable. Moreover, the text demonstrates a balanced integration of objective information and subjective opinions or judgments, indicating a well-maintained equilibrium between objective reporting and evaluative commentary on the campaign's limitations and impacts. The pharmacists highlighted gaps in awareness and the lack of direct impact on community pharmacies, emphasizing the need for better communication and more effective measures.

### 3.3 Theme 4: strategies to improve the rational use of antibiotics in community pharmacies

The main methods of antibiotic use in community pharmacies that the staff pharmacists suggested were the release of antibiotics: not requiring an insurance examiner to open the package of purchased antibiotics; freely available antibiotics; and antibiotics from a dealer in private pharmaceuticals. The other methods suggested by community pharmacists include public awareness and knowledge of the appropriate and inappropriate use of antibiotics, interactions between patients and community pharmacists, awareness of pharmacies, promotion of pharmacist educational strategies, and government strategies. However, public awareness and knowledge of the use of antibiotics were also the most common methods suggested by the pharmacists.

*Public education is important. This will have a greater impact than educating healthcare providers. Healthcare professionals have enough knowledge, but pressure is placed on patients to supply inappropriate antibiotics. [Pharmacist 14, female, 62 years old, 30 years*' *experience, rural]. Sentiment: Polarity score: 0.2, Subjectivity score: 0.6: This text reflects a favorable sentiment toward the effectiveness of public education over educating healthcare providers, acknowledging existing knowledge among professionals but highlighting patient pressure. The subjectivity score is moderately high, reflecting personal opinion and experience.*

Various ways to educate the public are recommended. These include routine patient education during pharmacy services; education via mass media, such as television, radio, and social media; and education through local community health workers. The messages that are used to communicate are also a key factor in the effectiveness of public education. The pharmacists suggested that the messages communicated to the general population should be concise and hence be more attractive to the public. The risks of antibiotics should be included.

*I think, the short, concise, and easy-to-understand message is important [to communicate with the public] because today is an online society, everything should be short, so it would [then] be interesting. [Pharmacist 5, female, 27 years old, 2 years*' *experience, urban] Sentiment: Polarity score: 0.25, subjectivity score: 0.5: This text reflects favorable sentiment toward the importance of clear and concise public messaging. The subjectivity score is moderate, balancing personal insight with practical recommendations.*

*Another issue is the reduction of misuse [of antibiotics]. We should explain the disadvantages [of antibiotics]. If a patient does not want it, we cannot force them to take it. [Pharmacist 21, female, 38 years old, 5 years*' *experience, rural] Sentiment: Polarity score: 0.15, subjectivity score: 0.6: This text reflects favorable sentiment toward reducing antibiotic misuse through patient education. The subjectivity score is moderately high, indicating a mix of personal beliefs and practical advice.*

Pharmacists stress the importance of the public accepting a pharmacist as a healthcare provider and should be willing to seek advice on a health issue rather than self-medicating without the input of a pharmacist. Raising awareness among the public to build trust between community pharmacists and the public should be performed. A number of pharmacists believe that acting professionally, such as routinely taking a patient history and routinely providing patient counseling while dealing with patients, should help reinforce the role of the pharmacist as a trusted health advisor. A rural pharmacist indicated that patients often rely heavily on their recommendations due to fewer healthcare options nearby, making them more likely to accept the pharmacist's advice when antibiotics are not recommended.

*Whenever the patient trusts us, we will work as professionals more easily. The problem happened early on [after opening the pharmacy] when people came to a pharmacy and asked for anything they wanted, but the time went by, I could act as a professional more easily [people believe the pharmacist*'*s advice more easily]. In the long-term, we should make pharmacies reliable for people in the community. [Pharmacist 6, female, 27 years old, 3 years*' *experience, rural] Sentiment: Polarity score: 0.3, subjectivity score: 0.7: This text reflects a favorable sentiment toward building trust with patients and establishing professional and reliable pharmacy practices. The subjectivity score is high, reflecting personal experiences and opinions.*

A small number of interviewees stated that pharmacists should always be bound by ethics, the discharge of moral and professional obligations. Thus, raising awareness among pharmacists of the professional duty of pharmacists to ensure appropriate antibiotic use should be a priority.

*This is not a legal requirement that you are prohibited from supplying antibiotics [to patients] or otherwise, you will be guilty of legal offenses. This is about the ethics of pharmacists. Pharmacists should balance professional and business activities. [Pharmacist 11, female, 36 years old, 5 years*' *experience, rural] Sentiment: Polarity score: 0.2, subjectivity score: 0.6: This text reflects favorable sentiment toward ethical considerations in balancing professional and business responsibilities. The subjectivity score is moderately high, reflecting personal values and beliefs.*

*The attitudes of pharmacists should be changed to comply with the profession and balance profession and business interests. [Pharmacist 22, male, 36 years old, 10 years*' *experience, rural] Sentiment: Polarity score: 0.2, subjectivity score: 0.6: This text reflects favorable sentiment toward changing attitudes among pharmacists to better balance professional and business interests. The subjectivity score is moderately high, reflecting a personal perspective on professional behavior.*

Continuous professional development/education to keep up to date with current practice guidelines and recommendations for antibiotics is also recommended to supply antibiotics appropriately. To update the current practices of pharmacists, appropriate means, including providing booklets or other hard copies of simple current clinical practice guidelines to community pharmacies or online, are suggested. More training courses and online articles tailored to support community pharmacists were asked from participants to help them update their knowledge.

*CPE (Continuing Pharmacy Education) is one method [to promote appropriate use of antibiotics]. We [Pharmacists] can read the article online and do the test. However, only a few topics related to antibiotic use in community pharmacies exist. For face-to-face training, I attended the training provided by the Community Pharmacy Association. There are only a few topics related to the rational use of antibiotics. [Pharmacist 6, female, 27 years old, 3 years*' *experience, rural] Sentiment: Polarity score: 0.1, subjectivity score: 0.5: This text reflects a slightly favorable sentiment toward the role of continuing pharmacy education in promoting rational antibiotic use but also highlights the need for more relevant topics. The subjectivity score is moderate, balancing personal experience with concrete information.*

The development and enforcement of laws and regulations regarding antibiotic prescription and supply, including dispensing against a prescription, are necessary for promoting the rational use of antibiotics. Pharmacists outlined a need to review and update the relevant laws. Restrictions to supply antibiotics from community pharmacies, such as reclassifying antibiotics as prescription-only medicines or withdrawing some or all antibiotics from pharmacies, are recommended. *Re*-enforcement of the antibiotic supply by licensed pharmacists from qualified pharmacies should be implemented.

The law should reclassify antibiotics. Antibiotics should be withdrawn from pharmacies. [Pharmacist 21, female, 38 years old, 5 years' experience, rural] Sentiment: Polarity score: 0.15, subjectivity score: 0.6: This text reflects favorable sentiment toward stricter regulatory measures for antibiotic supply. The subjectivity score is moderately high, reflecting strong personal beliefs.

*The government should enforce the law to control pharmacies where there are no pharmacists to provide pharmacy services. [Pharmacist 16, female, 28 years old, 5 years*' *experience, urban] Sentiment: Polarity score: 0.2, subjectivity score: 0.5: This text reflects favorable sentiment toward government enforcement of regulations to ensure the presence of pharmacists in pharmacies. The subjectivity score is moderate, reflecting both personal opinion and a call for action.*

Some pharmacists have suggested that controlling advertisements regarding the promotion of antibiotic sales should be implemented to reduce the supply of antibiotics.

*Pharmaceutical companies are also involved. The promotion of selling antibiotics to pharmacies should not be promoted. [Pharmacist 2, male, 49 years old, 15 years*' *experience, urban] Sentiment: Polarity score: Polarity score: 0.1, subjectivity score: 0.5: This text reflects a slightly favorable sentiment toward limiting pharmaceutical promotions to pharmacies. The subjectivity score is moderate, balancing personal viewpoints with regulatory suggestions.*

The motivation of community pharmacists to be involved in promoting antibiotic use campaigns by providing incentives for participating pharmacies was suggested. However, others thought that pharmacists should hopefully perceive the benefit of the project and might be willing to promote the rational use of antibiotics in the long term without any incentives.

*Pharmacists may not be very interested. However, if there is a compensation [monetary incentive], this will help to induce pharmacists to join [the project] [Pharmacist 18, female, 34 years old, 1 years*' *experience, urban] Sentiment: Polarity Score: 0.1, Subjectivity Score: 0.5: This text reflects a slightly favorable sentiment toward providing monetary incentives to encourage pharmacist participation. The subjectivity score is moderate, reflecting personal opinions on motivation.*

Finally, many pharmacists suggested promoting the rational use of antibiotics throughout the health system, including public and private hospitals, clinics, and pharmacies, to all healthcare professionals, including doctors, pharmacists, and nurses. They noted that AMR was not the responsibility of only one healthcare professional or patient.

*There are many parts related to the inappropriate use of antibiotics, the general population, healthcare providers, patients, and the use of antibiotics in animals. They are all causes of antimicrobial resistance. [Pharmacist 10, female, 41 years old, 5 years*' *experience, urban] Sentiment: Polarity score: 0.15, subjectivity score: 0.4: This text reflects a favorable sentiment toward recognizing the multifaceted causes of antimicrobial resistance. The subjectivity score is lower, indicating a more objective acknowledgment of the issue.*

The sentiment analysis of the text discussing strategies to enhance the rational use of antibiotics in community pharmacies yields a polarity score of 0.18 and a subjectivity score of 0.57. These findings suggest that the text predominantly conveys a subtly optimistic and pragmatic stance. This favorable polarity reflects a constructive viewpoint toward improving community-based antibiotic management. Furthermore, the moderate subjectivity score indicates a balanced approach within the text, prioritizing objective recommendations and concrete details over subjective opinions or judgments. This analysis highlights pharmacists' proactive suggestions and strategies to address the challenges of antibiotic misuse, emphasizing the importance of education, regulation, and professional ethics in promoting rational antibiotic use in community pharmacies.

## 4 Discussion

Our findings highlight the complex interplay of patient demand, commercial pressures, pharmacists' knowledge and attitudes, and regulatory challenges in both urban and rural contexts. The findings from the sentiment analysis provide insights into the attitudes and practices of pharmacists, highlighting both the challenges and opportunities in promoting rational antibiotic use.

These results are in accordance with those of previous studies (as discussed below).^35^ However, this is the first study to show that pharmacists' perceptions of the advantages of antibiotics are significantly associated with their willingness to supply antibiotics via sentiment analysis. Community pharmacists commonly perform triage and supply medicines to treat a range of illnesses together with the necessary advice. The results from a previous study revealed that pharmacists reported supplying antibiotics without an appropriate indication and supplying antibiotics for a shorter duration than recommended. The study also reported the factors contributing to the inappropriateness of antibiotic supply by Thai community pharmacists, including younger pharmacists, those with less experience, Pharm. D. graduate pharmacists, employee pharmacists and those who work in a chain pharmacy.^35^

### 4.1 Patient demands and commercial pressures

In examining the barriers to adhering to the rational use of antibiotics, the sentiment analysis indicated a high degree of subjectivity and an overall unfavorable sentiment. The pharmacists highlighted various pressures and misconceptions that lead to inappropriate antibiotic use, emphasizing the challenges of balancing professional responsibilities with practical constraints.

Patient demand for antibiotics has emerged as a significant barrier to rational antibiotic use. Many community pharmacists reported feeling pressured to supply antibiotics because of patients' previous successful experiences with antibiotics, advice from family and friends, and even direct requests influenced by internet information and illegal advertisements from pharmaceutical companies. This aligns with previous research indicating that patient expectations and demand significantly influence pharmacists' dispensing practices, particularly where antibiotics are dispensed without legitimate prescriptions in the study setting.^17^^,^^19^  Pressure from patients to supply antibiotics was reported by community pharmacists in several low- to middle-income countries, even when there was no medical indication.^22^^,^^33^^,^^39^, ^40^, ^41^, ^42^, ^43^ Furthermore, the business nature of pharmacy practices, where pharmacists aim to retain customers, often leads to a supply of antibiotics.^39^^,^^44^ Community pharmacists were keen to please their customers to keep them loyal to their pharmacies to maintain business. The fear of losing patients to other pharmacies influenced pharmacists to supply antibiotics at a patient's request.^25^^,^^33^^,^^45^ In urban areas, the pressure from patients and the influence of commercial interests appear to be more pronounced, with pharmacists feeling compelled to meet patient demands despite recognizing the potential for inappropriate use. In contrast, rural pharmacists often have closer relationships with their patients, which can both enhance trust and complicate their ability to refuse requests for antibiotics. This approach demonstrates a commitment to patient education that varies in effectiveness between urban and rural contexts.^46^^,^^47^ Therefore, reducing patient demand for antibiotics may decrease the inappropriate supply of antibiotics from community pharmacies.

The sentiment analysis of the provided text on the theme of pharmacists' practices regarding antibiotic supply revealed that the overall sentiment was slightly favorable and moderately subjective. The pharmacists demonstrated a careful and balanced approach to antibiotic supply, emphasizing patient education and responsible decision-making. A lack of knowledge and awareness regarding antibiotics and antimicrobial resistance among the public was perceived as a cause of the inappropriate use of antibiotics by community pharmacists in this study as well as in previous studies.^1^^,^^33^^,^^48^, ^49^, ^50^ Educational interventions targeted at patients and the public improve overall knowledge, and the rational use of antibiotics has been recommended by study participants and is consistent with recommendations by the WHO.^5^

### 4.2 Knowledge and attitudes of pharmacists

This study revealed that a lack of adequate knowledge among community pharmacists about appropriate antibiotic use and antimicrobial resistance (AMR) contributes to the irrational supply of antibiotics. Inadequate knowledge by community pharmacists might have contributed to the inappropriate supply of an antibiotic. Sometimes, pharmacists have difficulties differentiating between bacterial and viral infections, which may contribute to irrational antibiotic prescribing/dispensing.^51^ A lack of knowledge regarding infection differentiation and antibiotic treatments is also an important factor contributing to the irrational supply of antibiotics from community pharmacies in Thailand.^35^ The findings from the community pharmacist interviews revealed that some community pharmacists mentioned some inappropriate key symptoms/criteria as a basis for supplying antibiotics to their patients. For example, many pharmacists stated that they would supply antibiotics when patients with upper respiratory infection symptoms had severe sore throat, fever, or discolored nasal discharge or sputum, and the symptoms lasted longer than three to four days. However, according to the guidelines,^52^^,^^53^ these are not key symptoms for which antibiotics are indicated for patients with sore throat. This finding was similar to those of previous studies reporting that pharmacists believe that antibiotics help to cure symptoms faster.^30^^,^^33^^,^^54^^,^^55^ Therefore, improving knowledge and raising awareness among pharmacists regarding appropriate antibiotic use is recommended. Updated clinical guidelines for the treatment of infectious diseases should be provided regularly to community pharmacists. Moreover, more educational activities related to the rational use of antibiotics in community pharmacies for continuing pharmacy education (CPE) credits should be provided to help pharmacists update their knowledge.

### 4.3 Regulatory challenges

From the viewpoint of community pharmacists, the current campaigns were not well publicized, and there was a lack of incentives or penalties. The sentiment analysis of the text addressing the limitations of current campaigns to promote the rational use of antibiotics in community pharmacies revealed overall unfavorable views, which were relatively subjective in nature. The pharmacists highlighted gaps in awareness and the lack of direct impact on community pharmacies, emphasizing the need for better communication and more effective measures.

This study identified a significant gap in the enforcement of laws and regulations governing antibiotic supply. In Thailand, most antibiotics can be supplied from community pharmacies by a community pharmacist without a prescription. This contributes to relatively easy access to antibiotics from community pharmacies and may lead to overuse and inappropriate use of antibiotics. During the interviews, some community pharmacists suggested restricting the oversupply of some or all antibiotics from community pharmacies. This may be because community pharmacists believe that this method does not help reduce the supply of antibiotics from community pharmacies or that this may affect the pharmacy business. However, policies and regulations should be put in place to enforce appropriate access to medicines. A number of studies similarly stated that enforcing regulatory measures restricting access to antibiotics was important for reducing inappropriate use.^17^^,^^44^^,^^50^^,^^56^, ^57^, ^58^, ^59^, ^60^ Rigorous implementation of restrictions on the overall supply of antibiotics has been shown to be effective in reducing nonprescription antibiotic consumption in Brazil, Mexico, Chile and South Korea.^61^, ^62^, ^63^ Rigorous implementation of restrictions on the overall supply of antibiotics has been shown to be effective in reducing nonprescription antibiotic consumption in Brazil, Mexico, Chile and South Korea.^64^ The illegal supply of medicines by nonpharmacists and nonpharmacy was a concern of the pharmacists in this study and was identified as an important factor leading to the inappropriate supply of medicines from community pharmacies in previous studies.^55^^,^^65^, ^66^, ^67^ This finding may reflect the weak or inadequate enforcement of laws and regulations regarding medicine distribution and sales, which may contribute to easy access to and inappropriate use of antibiotics.^19^^,^^21^^,^^39^^,^^45^^,^^50^^,^^68^, ^69^, ^70^, ^71^ Therefore, stricter enforcement of legislation regarding the illegal supply of antibiotics by nonpharmacists and noncommunity pharmacies is important to improve the rational use of antibiotics in Thailand.

With respect to the role of community pharmacists in improving the rational use of antibiotics, this study revealed positive perceptions by community pharmacists toward the important role of community pharmacists in reducing antibiotic resistance. However, many pharmacies reported not having participated in antimicrobial stewardship (AMS) campaigns in Thailand and had never heard about these antimicrobial stewardship campaigns or did not know how to take part in such campaigns. This possibly reflects a lack of publicity about the campaigns to community pharmacies and/or pharmacists. In a study in London, just over 80 % of community pharmacists indicated that AMR awareness campaigns are important for educating members of the public; however, their motivation to take part in such campaigns, including patient counseling about antibiotic use, was lacking.^68^ Community pharmacists in Thailand need to be motivated to participate in AMS programs. Possible ways to encourage community pharmacists to participate in a campaign in Thailand include effective publicity of the campaign, support from professional pharmacy organizations, and the 10.13039/501100004397Ministry of Public Health and training events about AMS.

### 4.4 Strengths and limitations

To our knowledge, this is the first qualitative study in which sentiment analysis was conducted in Thailand to explore the views of community pharmacists toward antibiotic supply and AMR. This interview study with community pharmacists was the first qualitative study regarding antibiotic use and supply from community pharmacies conducted in Thailand. While the relatively small number of community pharmacists who participated in the interview study is a limitation, it is acceptable for a study of this nature. Additionally, recall bias may have occurred when respondents were answering questions, potentially leading to under- or overreporting of views compared with actual behavior, especially if those views were related to less recent experiences.

## 5 Conclusion

In this study, community pharmacists provide pharmacy services by taking a patient history and giving advice to the patient when they supply antibiotic. However, several barriers to the rational use of antibiotics by community pharmacists, including patient demand, commercial interests, a lack of knowledge and awareness among community pharmacists, antibiotic use and AMR, and inadequate laws and regulations on antimicrobial utilization and the ineffectiveness of the enforcement of regulations on antibiotic access, have been identified.

The most important antibiotic stewardship strategies used to address these challenges in community pharmacies in Thailand involve multiple interventions that can promote public education, where community pharmacists play a vital role in advising and raising awareness among the general population about AMR and the proper use of antibiotics. The proposed interventions include enhancing knowledge and awareness of the rational use of antibiotics and AMR among community pharmacists, restricting access to antibiotics and implementing stricter laws governing antibiotic dispensing. Pilot programs are recommended to test the effectiveness of these interventions, comparing them with current practices to assess their impact on antibiotic use in community pharmacies. Collaborative efforts involving key stakeholders—physicians, pharmacists, healthcare providers, patients, and policymakers—are essential for developing robust national AMS programs.

## CRediT authorship contribution statement

**Rojjares Netthong:** Writing – review & editing, Writing – original draft, Visualization, Validation, Software, Methodology, Formal analysis. **Sisira Donsamak:** Writing – review & editing, Writing – original draft, Visualization, Validation, Software, Resources, Project administration, Methodology, Investigation, Funding acquisition, Formal analysis, Data curation, Conceptualization. **Dai N. John:** Visualization, Validation, Supervision, Methodology, Funding acquisition, Conceptualization. **Ros Kane:** Writing – review & editing, Visualization, Validation. **Keivan Armani:** Writing – review & editing, Visualization, Validation.

## Declaration of competing interest

The authors declare that they have no known competing financial interests or personal relationships that could have appeared to influence the work reported in this paper.

The author is an Editorial Board Member/Editor-in-Chief/Associate Editor/Guest Editor for *[Exploratory research in clinical and social pharmacy]* and was not involved in the editorial review or the decision to publish this article.

## Figures and Tables

**Figure:**
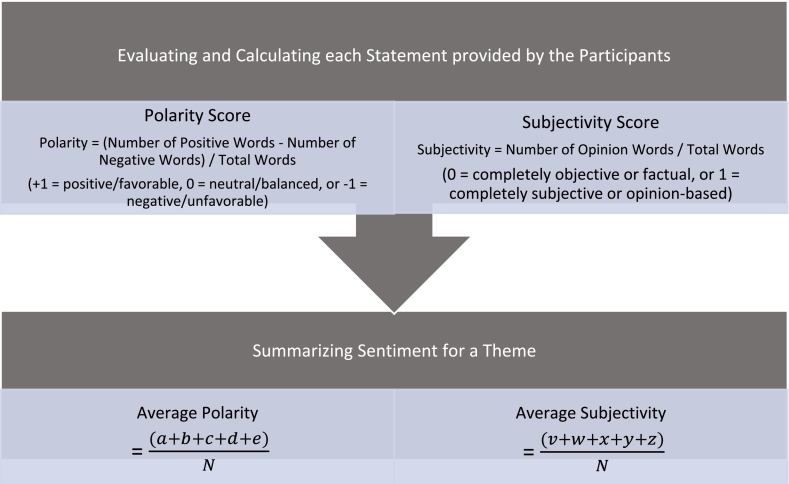
Fig. 1A Sentiment analysis process.

**Figure:**
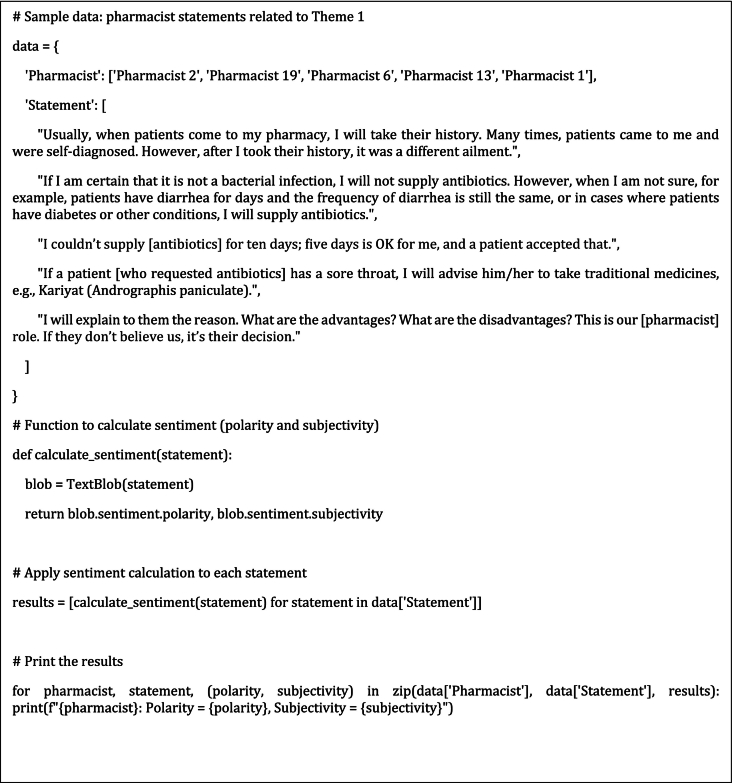
Fig. 2A Python code for sentiment analysis.

**Table:** Table 1A Polarity score and subjectivity score for each theme.

Pharmacist	Statement	Polarity Score	Subjectivity Score
**Theme 1: The practice of pharmacists regarding antibiotic supply**			
Pharmacist 2	“Usually, when patients come to my pharmacy, I will take their history. Many times, patients came to me and were self-diagnosed. However, after I took their history, it was a different ailment.”	0.1	0.6
Pharmacist 19	“If I am certain that it is not a bacterial infection, I will not supply antibiotics. However, when I am not sure, for example, patients have diarrhea for days and the frequency of diarrhea is still the same, or in cases where patients have diabetes or other conditions, I will supply antibiotics.”	0.05	0.7
Pharmacist 6	“I couldn't supply [antibiotics] for ten days; five days is OK for me, and a patient accepted that.”	0.1	0.65
Pharmacist 13	“If a patient [who requested antibiotics] has a sore throat, I will advise him/her to take traditional medicines, e.g., Kariyat (Andrographis paniculate).”	0.15	0.5
Pharmacist 1	“I will explain to them the reason. What are the advantages? What are the disadvantages? This is our [pharmacist] role. If they don't believe us, it's their decision.”	0.2	0.6
Average Polarity:		0.12	
Average Subjectivity:			0.61

**Theme 2: Barriers to comply with the rational use of antibiotics**			
Pharmacist 18	They had taken these medications [antibiotics], and they were cured, so they thought that if they were sick, they had to take them immediately.	−0.1	0.7
Pharmacist 4	When I ran my pharmacy for the first time, I felt like I wanted to solve the problem [inappropriate use of antibiotics]. However, when I faced real situations of pressure from patients and economics, I had to surrender in some cases.	−0.2	0.8
Pharmacist 14	For those who do not listen to me and insist on having it [antibiotics], I will supply it because if I don't supply them, they will visit another pharmacy. I need to keep them at my pharmacy.	−0.3	0.9
Pharmacist 13	It takes time to educate each patient and takes a long time. In the morning, I have multiple clients at once; I don't have time to educate each of them, so I have to supply what they want. If I take longer than five or ten minutes, the patients will rush me.	−0.25	0.8
Pharmacist 23	I think antibiotic resistance is not a big problem. They [other people] think that the inappropriate use of antibiotics from community pharmacies is one of the causes of serious bacterial resistance infections that are found in hospitals. It's totally different.	−0.15	0.7
Pharmacist 9	I think it is normal. Thus, antibiotics that have been used for a long period of time will become resistant. It's normal, but they [pharmaceutical companies] have been developing new antibiotics to fight resistant bacteria.	−0.1	0.6
Pharmacist 21	I usually ask patients about the duration of their illness and previous medications. If patients have sore throat, phlegm, or colored discharge, their symptoms last for 4–5 days, they have already taken other medicines, but they do not feel better, and I will supply antibiotics.	−0.05	0.5
Pharmacist 3	Antimicrobial resistance is not related to pharmacies. This is related to patients' behavior, which led to the old package of antibiotics and asked for them. AMR is a small problem. Pharmaceutical companies have already been preparing new antibiotics.	−0.2	0.7
Pharmacist 7	I think that supplying antibiotics from pharmacies is most appropriate. We [community pharmacists] supply only basic antibiotics for minor ailments. Inappropriate use of antibiotics, mostly from doctor clinics, is recommended.	−0.1	0.6
Average Polarity:		−0.18	
Average Subjectivity:			0.71

**Theme 3: Limitations of current campaigns to promote the rational use of antibiotics in community pharmacies**			
Pharmacist 4	I never heard about the current campaign. I heard about the promotion of using antibiotics appropriately only when I was studying [at the university].	−0.15	0.6
Pharmacist 21	There is no direct impact on the pharmacies [toward the appropriate or inappropriate supply of antibiotics]. For public health facilities, there are key performance indicators (KPIs), which are related to funding from the government.	−0.1	0.5
Average Polarity:		−0.13	
Average Subjectivity:			0.55

**Theme 4: Strategies to improve the rational use of antibiotics in community pharmacies**			
Pharmacist 14	Public education is important. This will have a greater impact than educating healthcare providers. Healthcare professionals have enough knowledge, but pressure is placed on patients to supply inappropriate antibiotics.	0.2	0.6
Pharmacist 5	I think, the short, concise, and easy-to-understand message is important [to communicate with the public] because today is an online society, everything should be short, so it would [then] be interesting.	0.25	0.5
Pharmacist 21	Another issue is the reduction of misuse [of antibiotics]. We should explain the disadvantages [of antibiotics]. If a patient does not want it, we cannot force them to take it.	0.15	0.6
Pharmacist 6	Whenever the patient trusts us, we will work as professionals more easily. The problem happened early on [after opening the pharmacy] when people came to a pharmacy and asked for anything they wanted, but the time went by, I could act as a professional more easily [people believe the pharmacist's advice more easily]. In the long-term, we should make pharmacies reliable for people in the community.	0.3	0.7
Pharmacist 11	This is not a legal requirement that you are prohibited from supplying antibiotics [to patients] or otherwise, you will be guilty of legal offenses. This is about the ethics of pharmacists. Pharmacists should balance professional and business activities.	0.2	0.6
Pharmacist 22	The attitudes of pharmacists should be changed to comply with the profession and balance profession and business interests.	0.2	0.6
Pharmacist 6	CPE (Continuing Pharmacy Education) is one method [to promote appropriate use of antibiotics]. We [Pharmacists] can read the article online and do the test. However, only a few topics related to antibiotic use in community pharmacies exist. For face-to-face training, I attended the training provided by the Community Pharmacy Association. There are only a few topics related to the rational use of antibiotics.	0.1	0.5
Pharmacist 21	The law should reclassify antibiotics. Antibiotics should be withdrawn from pharmacies.	0.15	0.6
Pharmacist 16	The government should enforce the law to control pharmacies where there are no pharmacists to provide pharmacy services.	0.2	0.5
Pharmacist 2	Pharmaceutical companies are also involved. The promotion of selling antibiotics to pharmacies should not be promoted.	0.1	0.5
Pharmacist 18	Pharmacists may not be very interested. However, if there is a compensation [monetary incentive], this will help to induce pharmacists to join [the project]	0.1	0.5
Pharmacist 10	There are many parts related to the inappropriate use of antibiotics, the general population, healthcare providers, patients, and the use of antibiotics in animals. They are all causes of antimicrobial resistance.	0.15	0.4
Average Polarity:		0.18	
Average Subjectivity:			0.57

## Appendix A Sentiment analysis process, code, and calculation

### A.1 Sentiment analysis process

Sentiment analysis was conducted to assess the emotional tone (polarity) and the objectivity/subjectivity (subjectivity score) of the responses given by the pharmacists. For each participant's statement, the following sentiment analysis metrics were applied, as shown in Fig. 1A below.

The polarity score is calculated by analyzing the number of positive/favorable versus negative/unfavorable words within the text. It ranges from −1 (most negative/unfavorable) to +1 (most positive/favorable). The subjectivity score measures how much of the text is based on personal opinions rather than factual information. It ranges from 0 (completely objective) to 1 (completely subjective). The following code was used to calculate the polarity and subjectivity scores for each statement provided by the pharmacists. The sentiment analysis was conducted using the TextBlob library in Python as a sample, as shown in Fig. 2A below.

### A.2 Sentiment calculation for each theme

The polarity and subjectivity of each pharmacist's statement related to each theme were calculated, as shown in Table 1A below.
